# Cost-Effectiveness Analysis of Fosfomycin for Treatment of Uncomplicated Urinary Tract Infections in Ontario

**DOI:** 10.1155/2017/6362804

**Published:** 2017-02-20

**Authors:** Louise Perrault, Sybil Dahan, Ange Christelle Iliza, Jacques LeLorier, George G. Zhanel

**Affiliations:** ^1^International Market Access Consulting, Montreal, QC, Canada; ^2^University of Montréal, Faculty of Medicine, Montréal, QC, Canada; ^3^Triton Pharma Inc., Concord, ON, Canada; ^4^Centre de Recherche du Centre Hospitalier de l'Université de Montréal, Montreal, QC, Canada; ^5^Department of Medical Microbiology and Infectious Diseases, University of Manitoba, Winnipeg, MB, Canada

## Abstract

*Background and Objective*. Bacterial resistance to antibiotics traditionally used to treat uncomplicated urinary tract infections (uUTIs) is rising in Canada. We compared the cost-per-patient in Ontario of including fosfomycin (an antibiotic with a low resistance profile) as an option for first-line empirical treatment of uUTIs with current cost of treatment with sulfonamides, fluoroquinolones, and nitrofurantoin.* Methods*. A decision-tree model was used to perform a cost-minimization analysis. All possible outcomes of a uUTI caused by bacterial species treated with either sulfonamides, fluoroquinolones, nitrofurantoin, or fosfomycin were included.* Results*. In the base case analysis, the cost-per-patient for treating uUTI with fosfomycin was $105.12. This is similar to the cost-per-patient for each of the other currently reimbursed antibiotics (e.g., $96.19 for sulfonamides, $98.85 for fluoroquinolones, and $99.09 for nitrofurantoins). The weighted average cost-per-patient for treating uUTI was not substantially elevated with the inclusion of fosfomycin in the treatment landscape ($98.41 versus $98.29 with and without fosfomycin, resp.). The sensitivity analyses revealed that most (88.34%) of the potential variation in cost was associated with the probability of progressing to pyelonephritis and hospitalization for pyelonephritis.* Conclusion*. Fosfomycin in addition to being a safe and effective agent to treat uUTI has a low resistance profile, offers a single-dose treatment administration, and is similar in cost to other reimbursed antibiotics.

## 1. Introduction

Urinary tract infections (UTIs) are common and, as such, are associated with substantial socioeconomic implications [1]. At least one-half of women report experiencing one or more UTIs in their lifetime [2, 3]. Most UTIs are uncomplicated bacterial infections of the lower urinary tract (uUTIs) [2, 4].* Escherichia coli* is the uropathogen responsible for 75% or more of uUTIs [5–8]. Many patients with uUTIs experience considerable impact on their health-related quality of life (HRQoL), including impairments in functioning at work, school, and home [9, 10]. Treatment of uUTIs is necessary not only to improve patient HRQoL, but also to reduce the risk of progression to pyelonephritis, a more serious condition [5].

Several antibiotics are used in Canada for treatment of uUTIs, including trimethoprim, trimethoprim–sulfamethoxazole (TMP–SMX), fluoroquinolones such as ciprofloxacin, nitrofurantoin, *β*-lactam antibiotics such as amoxicillin–clavulanate (AMC), and fosfomycin, a phosphoric acid derivative that represents its own class of antibiotics [11–14].

In addition to an already high burden of illness, standard treatment options are being challenged by rising bacterial resistance rates. Common uropathogens, in particular display variable resistance patterns over time and by region [5]. This requires practitioners to be aware of current local resistance rates and to be vigilant in tracking regional changes before empirically prescribing antibiotics for the treatment of UTIs [5]. For decades, TMP–SMX was considered first-line therapy for uUTIs [3], but rising rates of TMP–SMX resistance among uropathogens, and consistent evidence that in vitro TMP–SMX resistance correlates with bacterial and clinical failure, have forced a reappraisal of its first-line use for uUTIs [5]. Today, the* E. coli* resistance rate to TMP–SMX in Canada exceeds the 20% threshold at which guidelines recommend against TMP–SMX use for uUTI treatment [5, 11, 15–17].* E. coli* resistance to the commonly used fluoroquinolone ciprofloxacin was 19% in Canada in 2007–2009 [16], approximately twice the threshold above which the Infectious Diseases Society of America/European Society for Microbiology and Infectious Diseases (IDSA/ESCMID) clinical practice guidelines no longer recommend use of fluoroquinolones for uUTIs [5]. Bacterial resistance has several detrimental consequences for patients and society, including treatment failure and risk of complications, increased length of stay in hospital facilities, increased expenses, and increased mortality [18].

As bacterial resistance rates to current first-line antibiotics continue to rise, there will be a need for options that are effective, safe, and not likely to further promote community resistance. Although there is limited experience with fosfomycin for treating uUTIs in Canada [3, 19], it has been used for decades in other countries [20]. A meta-analysis of seven randomized controlled trials involving 1272 female patients with uUTIs showed no difference in cure rate between fosfomycin and a variety of comparators (including TMP–SMX, ciprofloxacin, and nitrofurantoin) [1]. Furthermore, the prevalence of* E. coli* resistance to fosfomycin is low (with an average resistance rate of 1.2% in a recent international survey) and has not shown a significant increase over time [21, 22].

To date, there is no published economic analysis that provides evidence on the relative cost-effectiveness of fosfomycin compared to recommended empirical therapy for uUTIs in Canada. The objective of this study was to examine the economic impact of using fosfomycin for first-line empirical treatment of uUTIs in adult women in Ontario, taking into account current antibiotic prices, treatment algorithms, event probabilities, and resistance rates.

## 2. Methods

### 2.1. Decision-Tree Model

Based on evidence of equivalent efficacy and safety in head-to-head trials [38–43] and a meta-analysis [1], we conducted a cost-minimization analysis (CMA) using a decision-tree model (TreeAge Pro 2015 software®, Williamstown, MA, USA). The model is based on a previous model of uUTI treatment caused by* E. coli* in women older than 18 years [27] and uses a payer perspective, specifically that of the Ontario Ministry of Health and Long-term Care. Only direct medical care costs are considered. The time horizon of the model included 2 treatment courses.

The model assumed that first-line empirical treatment of uUTIs would consist of one of the antibiotics currently reimbursed by the Ontario Drug Benefit Program (ODBP) [44] and recommended by recent guidelines [5, 13, 14] for the treatment uUTIs: sulfonamides (TMP/SMX DS 800 mg/160 mg, TMP 100 mg, or TMP 200 mg), fluoroquinolones (Cipro® XL™ 500 mg, ciprofloxacin 250 mg [generic], or norfloxacin 400 mg), or nitrofurantoins (MacroBID® 100 mg, nitrofurantoin 50 mg [generic], or nitrofurantoin 100 mg [generic]).

**Figure 1:**
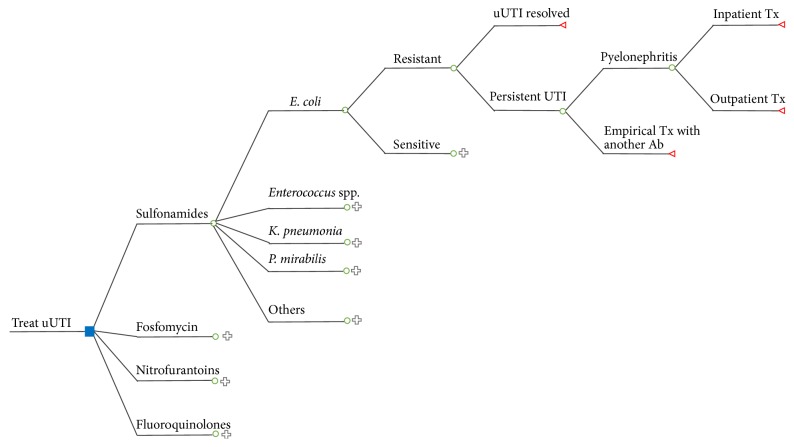
Decision-tree diagram showing intermediate and final outcomes for an uncomplicated* E. coli* UTI treated with a sulfonamide.

The decision-tree model incorporates all possible outcomes of a uUTI caused by* E. coli*,* Enterococcus* species,* Klebsiella pneumoniae*,* Proteus mirabilis*, or all other bacterial species (combined) treated with either fluoroquinolones, sulfonamides, nitrofurantoin, or fosfomycin (Figure 1). For simplicity, the model does not include the probability of rare complications associated with specific antibiotics (e.g., Stevens-Johnson syndrome). The probability of vaginal yeast infections is also not included because this complication is generally treated with over-the-counter drugs that are not reimbursed by the ODBP.

The model compares all four treatments and considers the possibility that the bacterial species would be susceptible or resistant to each antibiotic. In the latter case, the UTI either spontaneously resolves or persists. Patients with persistent UTIs either progress to pyelonephritis (with outpatient or inpatient treatment) or, in the absence of pyelonephritis, are treated with a different antibiotic.

The model was internally validated. All codes and programming were validated by a third party who was not involved in creating the model.

### 2.2. Costs

Province-level data on drug costs were used in the model. For the base case scenario, the cost of treatment for each antibiotic used to treat uUTIs was computed based on prices in the Ontario Drug Benefit Formulary (ODBF) [44] and the recommended dosage for uUTIs, as outlined by recent clinical guidelines [5, 13, 14]. The cost of each comparator was obtained by calculating an average cost of treatment for the class of antibiotic weighted by the volume of claims reimbursed by the ODBP as reported by Pharmastat data (Table 1).

Data on healthcare resource use were obtained from multiple public sources including the Physician Services under the Health Insurance Act (April 1, 2016), Ontario Case Costing Initiative, and from Pharmastat provincial data [24, 45]. Healthcare resource use varied by outcome. For uUTIs that resolved with first-line empirical treatment, a single clinic visit was included in the model. Urinalysis and urine cultures were not performed prior to first-line treatment. For uUTIs that persisted and required treatment with another antibiotic, the model included two clinic visits (one initial visit and one following failure of the first-line antibiotic) and a urine culture. Progression to pyelonephritis was assumed to occur during or after first-line treatment; thus, patients with pyelonephritis requiring outpatient treatment consumed the following resources: one initial clinic visit, one emergency room visit, two follow-up clinic visits (one 48 hours after initiation of treatment for pyelonephritis and one at the end of treatment), and one urine culture at the end of treatment to confirm resolution of infection. For patients with pyelonephritis requiring inpatient treatment, the model included an initial clinic visit, hospitalization for pyelonephritis, and a follow-up clinic visit with a urine culture at the end of treatment to confirm resolution of infection.

First-line empirical treatment could be any one of the following: sulfonamide, fluoroquinolone, nitrofurantoin, or fosfomycin. Second-line treatment for persistent infections was either Cipro® XL™ 500 mg once per day for 3 days (if first treatment was not ciprofloxacin) or MacroBID® 100 mg twice per day for 7 days (if first treatment was ciprofloxacin). Pyelonephritis was treated with either Cipro® XL™ 500 mg/1 g once per day for 7 days (if first treatment was not ciprofloxacin) or Clavulin® 500/125 g three times per day for 14 days (if first treatment was ciprofloxacin).

Costs associated with productivity loss are not included in the analysis; only direct medical care costs are considered.

### 2.3. Probabilities

Probabilities were obtained from published estimates. Probabilities of resistance for fluoroquinolones, sulfonamides, and nitrofurantoin were obtained from the CANWARD surveillance study 2007–2011 [15] and the CANWARD surveillance study 2007–2009 (Table 2) [16]. Resistance rates for fosfomycin were obtained from the ARESC (Antimicrobial Resistance Epidemiological Survey on Cystitis) study [22]. Probabilities for effectiveness were assumed to be equal for all antibiotics based on the meta-analysis published by Falagas et al. (2010) [1]. Effectiveness was assumed to be reduced by 20% for resistant isolates [33]. Probabilities of pyelonephritis (i.e., pyelonephritis due to initial treatment failure and hospitalization for pyelonephritis) were obtained from different sources [27, 28, 34–36].

### 2.4. Sensitivity Analyses

Variables suspected to have the most influence on the expected value of cost-per-patient were subjected to a deterministic one-way sensitivity analysis (DSA). Where possible, ranges for variables were extracted from the literature or other public sources. In the absence of a reliable source, a hypothesis of the plausible range was used. The base case values and the ranges tested in the sensitivity analyses are shown in Tables 1 and 2.

## 3. Interpretation

This study uses a cost-minimization analysis (CMA) to identify the most affordable options for the treatment of uUTI in Ontario, taking into account both cost-per-dose and costs associated with antibiotic resistance.

### 3.1. Analysis and Results

**Figure 2:**
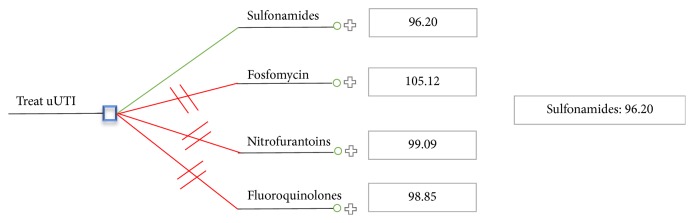
Cost-per-patient for treating uUTI in Ontario.

With the current cost of fosfomycin of $17.51, the cost-per-patient for treating uUTIs with this drug was determined to be $105.12. This is comparable to the cost-per-patient for each of the other currently reimbursed antibiotics (Figure 2).

**Figure 3:**
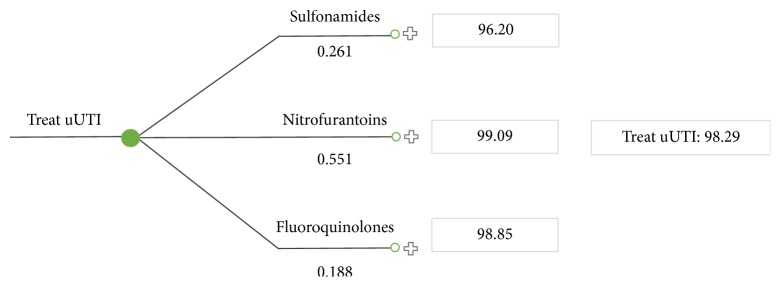
Weighted average cost-per-patient for treating uUTI in Ontario without fosfomycin.

The weighted average cost-per-patient for treating uUTI in Ontario was also estimated based on 2 scenarios: with and without fosfomycin. In this analysis, cost-per-patient was obtained by replacing the decision node of the tree with a chance node which identifies probabilities of events. The probabilities were the market share of each alternative treatment. Thus, the weighted average cost takes into account the antibiotics currently reimbursed by the ODB and their respective market shares. The weighted average cost-per-patient for treating uUTI in Ontario is $98.29 (Figure 3). Market shares are based on Ontario Pharmastat data.

**Figure 4:**
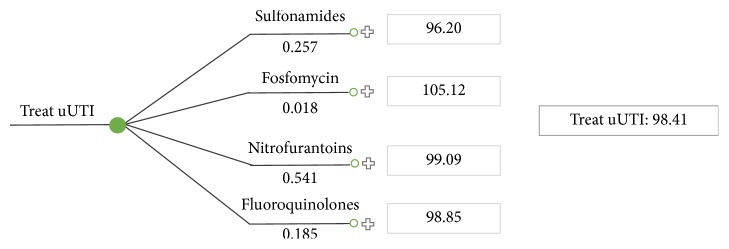
Weighted average cost-per-patient for treating uUTI in Ontario with fosfomycin.

Incorporating fosfomycin into the mix of treatments reimbursed in Ontario results in a weighted average cost-per-patient of $98.41 (Figure 4). For this analysis, the estimated market share of fosfomycin was 1.77% as reported by Pharmastat. The impact of reimbursing fosfomycin in the Ontario treatment landscape has a negligible impact on the weighted cost-per-patient and provides physicians with an effective alternate treatment option.

### 3.2. Results of Uncertainty Analysis

**Figure 5:**
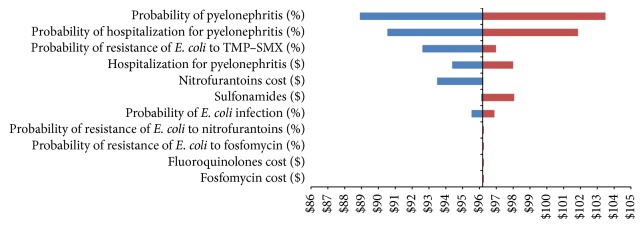
Tornado diagram comparing the results of the one-way sensitivity analyses of variables potentially affecting the expected value of the cost-per-patient (decision node).

Sensitivity analyses were performed based on the decision and chance nodes of the decision tree. The tornado diagram revealed that the probability of developing pyelonephritis has the largest potential effect on the expected value (cost-per-patient) of the model (Figure 5). Other variables that had a potential effect on cost-per-patient were as follows:

- Probability of hospitalization for pyelonephritis
- Probability of resistance of* E. coli* to TMP–SMX
- Cost of hospitalization for pyelonephritis
- Cost of nitrofurantoin
- Probability of being infected by* E. coli*

Variables accounting for 93.18% of the potential variation in cost-per-patient were the probability of progressing to pyelonephritis (55.13%), having to be hospitalized for pyelonephritis (33.17%), and contracting an infection with TMP–SMX-resistant* E. coli *(4.87%). Detailed results of the one-way sensitivity analyses are presented in Appendix.

**Figure 6:**
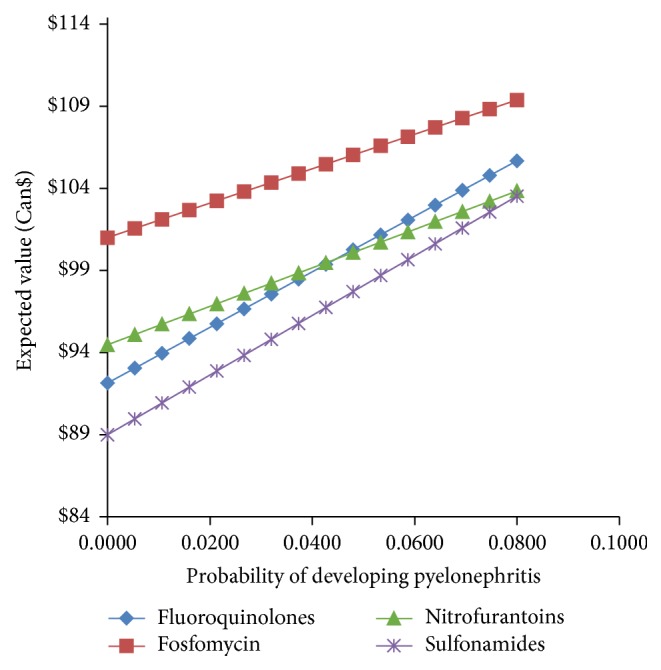
Comparison of the expected value of the cost-per-patient when treating uUTI with fluoroquinolones, sulfonamides, nitrofurantoin, and fosfomycin versus the probability of developing pyelonephritis.

Figure 6 compares the cost-per-patient for treating uUTI with a fluoroquinolone, sulfonamide, nitrofurantoin, or fosfomycin with various probabilities of progression to pyelonephritis. At all levels of risk of progression to pyelonephritis, fosfomycin is associated with the highest cost-per-patient. As the risk of pyelonephritis increases, the expected values of other antimicrobial treatments approach that of fosfomycin. Note that the base case value for progression to pyelonephritis is 4% [27, 28, 34, 35].

**Figure 7:**
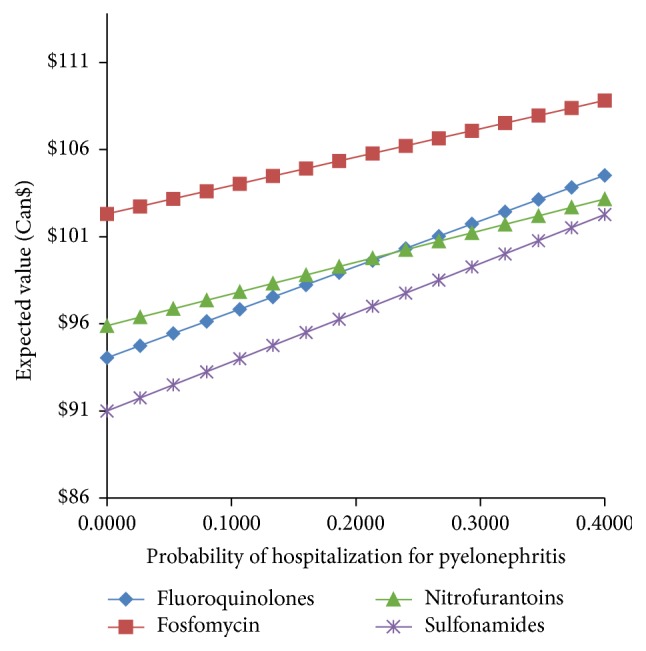
Comparison of the expected value of the cost-per-patient when treating uUTI with fluoroquinolones, sulfonamides, nitrofurantoin, and fosfomycin versus the probability of hospitalization for pyelonephritis.

Figure 7 compares the cost-per-patient for treating uUTI with one of the 4 antimicrobial treatments with the probability of hospitalization due to pyelonephritis. At all probabilities, fosfomycin is associated with the highest cost. Note that the base case value for the probability of hospitalization due to pyelonephritis is 20% [36].

**Figure 8:**
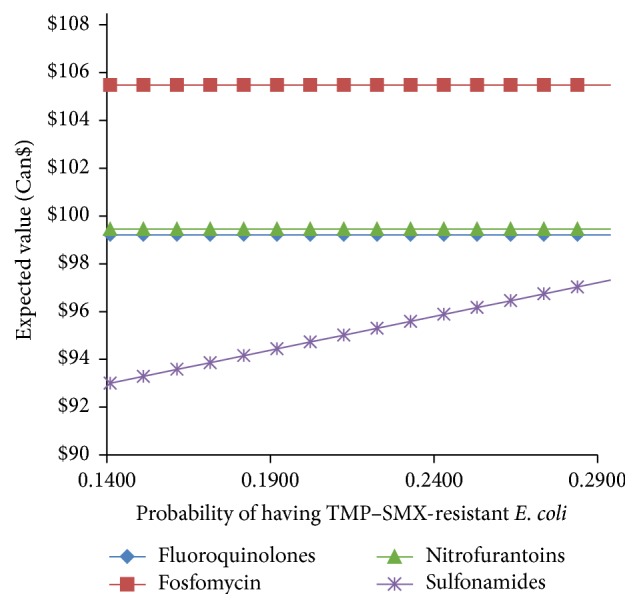
Comparison of the expected value of the cost-per-patient when treating uUTI with fluoroquinolones, sulfonamides, nitrofurantoin, and fosfomycin versus the probability of a uUTI with TMP–SMX-resistant* E. coli*.

Figure 8 compares the expected cost-per-patient for the treatment of a uUTI with a fluoroquinolone, sulfonamide, nitrofurantoin, or fosfomycin with the prevalence of TMP–SMX-resistant* E. coli*. The cost-per-patient when using a sulfonamide steadily increases with increasing resistance probabilities. The cost-per-patient of the other antibiotics is not affected by this variable. Note that the base case value for* E. coli* resistance to sulfonamides is 26.7% [15].

**Figure 9:**
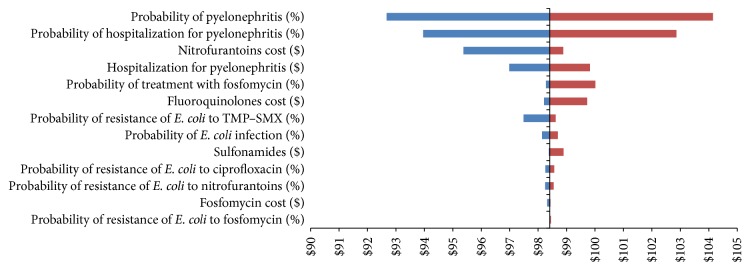
Tornado diagram comparing the results of the one-way sensitivity analyses of variables potentially affecting the expected value of cost-per-patient (chance node).

A tornado diagram was also developed using the one-way sensitivity analyses of the chance node instead of the decision node of the decision tree (Figure 9). The major difference in the results of this sensitivity analysis was that the cost of nitrofurantoins was the third highest contributor to the potential variation in cost-per-patient—meaning that the cost of nitrofurantoins has a great influence on the cost of treating patients with uUTI covered by the ODB. Recall that, in the decision node analysis, the third highest contributor was probability of contracting an infection with TMP–SMX-resistant* E. coli*.

## 4. Discussion

Although there is limited experience with fosfomycin for treating uUTI in Canada [3], it has been used successfully for decades in other countries [19, 20]. Fosfomycin is a phosphoric acid derivative that represents its own class of antibiotics; no other member of this class is currently approved by regulatory agencies worldwide [19, 20].

Resistance rates for ciprofloxacin and TMP–SMX (INESSS first-line recommendations for treatment of uUTI) were much lower a decade ago than they are now. The ECO-SENS survey, conducted in 1999-2000, showed* E. coli* resistance rates of 0% for ciprofloxacin and 12% for TMP–SMX in Canada (*n* = 166, urinary isolates) [6]. As of 2009, the CANWARD surveillance initiative revealed that* E. coli* resistance to TMP–SMX in urinary isolates was 24.3% and had undergone a significant increase over just a 3-year period—the resistance rate was 18.6% in 2007 (data derived from urine samples of 2943 outpatients and inpatients, *P* = 0.02) [16]. Recent data from the CANWARD surveillance initiative (2011) focusing on resistance rates regardless of source (i.e., not specific to uropathogens) revealed that* E. coli* resistance to ciprofloxacin and TMP–SMX is quite high in Canada (26.9% for ciprofloxacin and 29.3% for TMP–SMX, *N* = 646). Ontario resistance rates in 2011 were similar to those seen across Canada (24.6% for ciprofloxacin and 28.1% for TMP–SMX, *N* = 167) [46]. It is clear that antibiotic resistance is on the rise, particularly for ciprofloxacin and TMP–SMX.

In contrast, the ECO-SENS survey (conducted in 1999-2000 and then again in 2007-2008) showed that the initial low resistance rate of* E. coli* to fosfomycin did not increase significantly over time. In* E. coli* isolates from 5 European countries, the resistance rate to fosfomycin was 0.4% in 1999-2000 and 1.2% in 2007-2008 [21]. This consistently low resistance rate may be due to the fact that fosfomycin resistance comes at a biological cost to microorganisms [19]. Furthermore, the low resistance of* E. coli* in the ECO-SENS survey could be explained by the exclusion of hospitalized patients during the two weeks before the onset of symptoms and data from the CANWARD surveillance initiative were based on samples from outpatients and inpatients. Microbiological studies have shown that fosfomycin-resistant strains of* E. coli* are “biologically impaired”—they take up to 39% longer to replicate and they have a 38% reduction in the ability to adhere to uroepithelial cells. These resistant strains are unable to compete with susceptible strains in the environment, possibly contributing to the low rates of fosfomycin resistance observed over time [19, 47].

Fosfomycin also has a low propensity for cross-resistance, which is likely due to the fact that it differs from other antibiotics in its general chemical structure and site of action [19, 48]. The unique mechanism of action of fosfomycin appears to underlie its low rate of cross-resistance. This mechanism of action, which inhibits the early stages of cell wall synthesis [49], reduces the risk of cross-resistance with other antibacterial agents, making fosfomycin a good choice for the treatment of multidrug-resistant strains and leaving options available for further therapies [19, 47]. In the ECO-SENS survey, cross-resistance in* E. coli* was observed with ampicillin and SMX, alone or linked with resistance to TMP and TMP–SMX. A multiple-resistant phenotype involving fluoroquinolone resistance was also noted. The ECO-SENS authors concluded that resistance to any agent was associated with increased resistance to all other agents tested, fosfomycin being the only exception [50].

In addition to the biological aspect of the low fosfomycin resistance rate, single-dose antibiotics are associated with a high degree of compliance and greater convenience, which may help prevent resistance from developing [19, 51, 52]. Maintenance of 100% adherence to optimized dosing regimens is critical for ensuring antimicrobial efficacy of the drug and prevention of resistance. Experts have recommended that underdosing be prevented to “suppress or decrease the potential amplification of resistant mutant subpopulations” and that regimens should be as short as possible in order to maximally suppress resistance [52]. Other potential benefits of single-dose therapy include directly observed therapy (i.e., compliance is not an issue) and fewer side effects [53].

Since resistance patterns of* E. coli* strains causing uUTI vary considerably across regions and can be well beyond the resistance threshold for certain antibiotics, the IDSA/ESCMID guidelines note that a specific treatment recommendation may not be universally suitable [5, 46]. The guidelines now include fosfomycin as one of the recommended first-line options for the treatment of uUTIs along with nitrofurantoin, TMP–SMX, and pivmecillinam [5]. In May 12, 2016, the FDA made recommendations resulting from a November 5, 2015, meeting, (Joint Meeting of the Antimicrobial Drugs Advisory Committee [formerly known as the Anti-Infective Drugs Advisory Committee] and Drug Safety and Risk Management Advisory Committee Meeting) that, based on risk versus benefit, fluoroquinolones (such as ciprofloxacin and levofloxacin) should not be used first line for the treatment of uUTI; rather nitrofurantoin, TMP–SMX, and fosfomycin should be used instead [54].

In this new world of rapidly advancing microbial resistance, an additional antibiotic in the armamentarium, especially one with a low resistance profile, may be of substantial benefit to Ontario practitioners and patients.

### 4.1. Summary of Results

This analysis revealed that the cost-per-patient for treating uUTI in Ontario is similar for all 4 antibiotics, ranging from $96.19 for sulfonamides to $105.12 for fosfomycin. The average cost-per-patient for treating uUTI in Ontario with all 4 reimbursed antimicrobial agents (fosfomycin, fluoroquinolones, sulfonamides, or nitrofurantoin), weighted by market share, is $98.41 (versus $98.29 without fosfomycin).

Most of the potential variability in cost-per-patient (93.18%) is associated with the probability of progressing to pyelonephritis (55.13%), having to be hospitalized for pyelonephritis (33.17%), and contracting an infection with TMP–SMX-resistant* E. coli* (4.87%).

### 4.2. Study Limitations

There were limitations to this study. First, efficacy was assumed to be equal based on the meta-analysis conducted by Falagas et al. (2010) [1]. However, this analysis was only focused on comparing fosfomycin with other antibiotics. Meta-analyses providing efficacy comparisons between the other antimicrobial agents were not identified in the literature. Similarly, we were unable to locate sources for the efficacy of each agent in susceptible versus resistant infections.

Secondly, the only published data we had for the probability of developing pyelonephritis was from a US source. As the model was the most sensitive to this variable, we cannot definitively determine its reliability in Canada.

### 4.3. Generalizability

This model has been constructed to specifically address the empirical treatment of uUTI in women older than 18 years of age. This population is aligned with the current approved indication of fosfomycin in Canada.

The data used to populate the model were derived from Ontario and/or Canadian sources wherever possible. Costs and market share of currently reimbursed antibiotics were derived from provincial data, providing an accurate view of the present market.

Consultation with experts on clinical practice patterns and costs provided an additional layer of real-world validity.

### 4.4. Health Services Impact

Since the availability of fosfomycin does not significantly increase the weighted average cost of treatment per patient, reimbursement of this antibiotic in Ontario has a negligible effect on the provincial healthcare budget. The societal implications are, however, substantial. Providing access to an additional antibiotic, especially one with a low resistance profile like fosfomycin, may benefit both Ontario practitioners and their patients. With rising rates of resistance to fluoroquinolones and sulfonamides, these “staple” antibiotics are no longer broadly considered appropriate first-line empirical therapies by international experts [5]. This shift of recommended treatments in the armamentarium leaves physicians in Ontario with fewer options for treating uUTIs. Reimbursement of fosfomycin by the ODBP would allow physicians to effectively treat patients with uUTIs while ensuring that community resistance to important antibiotics (fluoroquinolones and sulfonamides) is not further increased in Ontario.

### 4.5. Future Research

Further research into the efficacy of fluoroquinolones, sulfonamides, and nitrofurantoin in resistant infections would be beneficial for future pharmacoeconomic evaluations, especially since the rates of resistance for some of these agents have risen drastically in a short period of time. Additionally, head-to-head studies of antibiotic efficacy and safety in current uUTI patient populations would provide much needed data for developing accurate probabilities of treatment success/failure.

## 5. Conclusions

Fosfomycin in addition to being a safe and effective agent to treat uUTI has a low resistance profile, offers a single-dose treatment regimen, and is comparable in cost to other reimbursed antibiotics.

## Figures and Tables

**Table 1:** Cost of antibiotics and healthcare resources used for the treatment of uUTIs and the ranges tested in the sensitivity analyses.

Variable	Base case (CAD $)	Range tested (CAD $)	References
Fosfomycin	17.51	13.00–18.00	[23], Pharmastat
Sulfonamides	0.95	0.88–2.79	[23], Pharmastat
Fluoroquinolones	4.25	3.71–11.78	[23], Pharmastat
Nitrofurantoin	10.54	4.68–11.12	[23], Pharmastat
Hospitalization for pyelonephritis	6,918.15	4,842.71–8,993.60	OCCI, hypothesis of plausible range (±30% of base case)
Initial clinic visit	77.20	—	[24]
Follow-up clinic visit	45.90	—	[24]
Urine culture	45.00	—	Laboratoires Biron, oral communication, May 30 2016
Emergency visit	287.60^a^	—	[24–26]

__^a^Emergency MD cost ($97.60) + total cost for an emergency visit in Quebec in 2009, MD cost excluded ($190).

**Table 2:** Probability values used in the model for decision-tree nodes and ranges used in the sensitivity analyses.

Variable	Base case	Range tested	References
Sensitive isolate: UTI resolution (clinical cure)	0.94	—	[27–32]
Resistant isolate: UTI resolution (clinical cure)	0.74	—	[33]
Pyelonephritis due to initial treatment failure	0.04	0–0.08	[27, 28, 34, 35], hypothesis of plausible range
Hospitalization for pyelonephritis	0.20	0–0.40	[36], hypothesis of plausible range
Empirical extended treatment due to initial treatment failure (switch from initial treatment to another antibiotic)	0.75/0.25 (0.25/0.75 for ciprofloxacin)	—	[27, 28, 35]
Pathogens present in UTI			
*E. coli*	0.841	0.767–0.918	[7, 22, 37]
*Enterococcus spp.*	0.028	—	[37]
*K. pneumoniae*	0.038	—	[37]
*P. mirabilis*	0.026	—	[37]
Treatment with fosfomycin	0.0177	0–0.250	Pharmastat, hypothesis of plausible range
Treatment with sulfonamides	0.2566	—	Pharmastat
Treatment with nitrofurantoins	0.5410	—	Pharmastat
Treatment with fluoroquinolones	0.1848	—	Pharmastat
*E. coli* resistance to fosfomycin	0.006	0.004–0.012	[6, 21, 22]
*E. coli* resistance to sulfonamides (TMP–SMX)	0.267^a^	0.141–0.294	[15, 16]
*E. coli* resistance to fluoroquinolones (ciprofloxacin)	0.218^a^	0.191–0.245	[6, 15, 22]
*E. coli* resistance to nitrofurantoin	0.011^a^	0.0016–0.019	[15, 16, 22]

TMP–SMX, trimethoprim–sulfamethoxazole; UTI, urinary tract infection.

__^a^Derived from urinary, respiratory, wound, and blood isolates.

**Table 3:** Results of tornado diagram (decision node).

Variable	Range	Low EV	High EV	Spread	Spread^2^	Risk (%)	Cumulative risk (%)	Variable index
Probability of developing PN	0.0 to 0.08	88.93	103.46	14.530	211.121	0.553	0.553	0
Probability of hospitalization for PN	0.0 to 0.4	90.56	101.83	11.270	127.013	0.332	0.885	1
Probability of resistance of *E. coli* to TMP–SMX	0.141 to 0.294	92.64	96.96	4.320	18.662	0.049	0.934	2
Cost of hospitalization for PN	4842.71 to 8993.60	94.41	97.98	3.570	12.745	0.033	0.967	3
Cost of nitrofurantoin	4.68 to 11.12	93.51	96.20	2.690	7.236	0.019	0.986	4
Probability of being infected by *E. coli*	0.767 to 0.918	95.56	96.86	1.300	1.690	0.004	1	5
Probability of resistance of *E. coli* to nitrofurantoin	0.0016 to 0.019	96.20	96.20	0.000	0.000	0.000	1	6
Probability of resistance of *E. coli* to fosfomycin	0.004 to 0.012	96.20	96.20	0.000	0.000	0.000	1	7
Probability of resistance of *E. coli* to ciprofloxacin	0.191 to 0.245	96.20	58.69	0	0	0.000	1	8
Cost of ciprofloxacin	3.71 to 11.78	96.20	96.20	0.000	0.000	0.000	1	9
Cost of fosfomycin	13.0 to 18.0	96.20	58.69	0	0	0.000	1	10

EV, expected value; PN, pyelonephritis.

## Appendix

See Table 3.
